# MR Diffusion Tensor Imaging Detects Rapid Microstructural Changes in Amygdala and Hippocampus Following Fear Conditioning in Mice

**DOI:** 10.1371/journal.pone.0051704

**Published:** 2013-01-30

**Authors:** Abby Y. Ding, Qi Li, Iris Y. Zhou, Samantha J. Ma, Gehua Tong, Grainne M. McAlonan, Ed X. Wu

**Affiliations:** 1 Laboratory of Biomedical Imaging and Signal Processing, The University of Hong Kong, Hong Kong SAR, China; 2 Department of Electrical and Electronic Engineering, The University of Hong Kong, Hong Kong SAR, China; 3 Department of Psychiatry, The University of Hong Kong, Hong Kong SAR, China; 4 Centre for Reproduction Growth and Development, The University of Hong Kong, Hong Kong SAR, China; 5 Department of Anatomy, The University of Hong Kong, Hong Kong SAR, China; 6 Department of Forensic and Neurodevelopmental Science, Institute of Psychiatry, King’s College London; University of Florida, United States of America

## Abstract

**Background:**

Following fear conditioning (FC), ex vivo evidence suggests that early dynamics of cellular and molecular plasticity in amygdala and hippocampal circuits mediate responses to fear. Such altered dynamics in fear circuits are thought to be etiologically related to anxiety disorders including posttraumatic stress disorder (PTSD). Consistent with this, neuroimaging studies of individuals with established PTSD in the months after trauma have revealed changes in brain regions responsible for processing fear. However, whether early changes in fear circuits can be captured *in* *vivo* is not known.

**Methods:**

We hypothesized that *in vivo* magnetic resonance diffusion tensor imaging (DTI) would be sensitive to rapid microstructural changes elicited by FC in an experimental mouse PTSD model. We employed a repeated measures paired design to compare *in vivo* DTI measurements before, one hour after, and one day after FC-exposed mice (n = 18).

**Results:**

Using voxel-wise repeated measures analysis, fractional anisotropy (FA) significantly increased then decreased in amygdala, decreased then increased in hippocampus, and was increasing in cingulum and adjacent gray matter one hour and one day post-FC respectively. These findings demonstrate that DTI is sensitive to early changes in brain microstructure following FC, and that FC elicits distinct, rapid *in vivo* responses in amygdala and hippocampus.

**Conclusions:**

Our results indicate that DTI can detect rapid microstructural changes in brain regions known to mediate fear conditioning *in vivo*. DTI indices could be explored as a translational tool to capture potential early biological changes in individuals at risk for developing PTSD.

## Introduction

Posttraumatic stress disorder (PTSD) can occur in persons who experience an intensely fearful event. Although individual symptoms of PTSD are present to a variable extent in almost all people in the days and weeks following trauma exposure [1], only some will develop full-blown PTSD, which is defined by sustained symptoms for more than one month following exposure [2]. Early treatment of those at risk for PTSD prior to development of the chronic disorder has been proven to be effective [3]. However, such treatment opportunity is greatly limited by the lack of an established biomarker for PTSD [4]. To date, a few potential biomarkers have been suggested, including ‘p11’ which may be measured using a blood test [5], magnetoencephalography indices (MEG) [6], as well as response in a conditional discrimination paradigm [7].

Neuroimaging studies have found that the brain of patients with PTSD is structurally and functionally different from unaffected control individuals [8]. It is thought that changes in cellular morphology and accompanying alterations in brain volume contribute to this neural plasticity or remodeling that underlies the onset of PTSD [9]. However, human studies have been complicated by heterogeneous findings, most likely due to the complexity of individual past experiences and variable individual physiological baseline [8], [10]. Thus, animal models of PTSD, which are considered to have face and construct validity for aspects of this complex clinical disorder, enable a ‘proof of principle’ study under experimental conditions [11], [12] and there are many PTSD-specific models available to reproduce aspects of PTSD [13]. Models involving inescapable shock have been demonstrated to produce a range of behavioral and biological characteristics with similarities to PTSD [14], [15], [16], [17]. Among the various paradigms involving inescapable shock, classical fear conditioning (FC) has been widely employed. It has been proposed that the main clinical features of PTSD, including re-experiencing phenomena, avoidance and hyperarousal, might reflect strong associative learning akin to FC [18], [19].

The neurobiology of FC in terms of its anatomical, functional, and molecular pathways is well documented [20], [21]. Specifically, FC depends on neurocircuitry incorporating amygdala and hippocampal projections [10], [22], [23], [24]. The creation of stable and persistent long-term fear memory not only requires gene expression and the resultant synthesis of new proteins but also involves structural changes in synaptic morphology in these key brain regions [25], [26], [27], [28], [29]. Persistent synaptic changes, rapidly mediated by cytoskeletal molecules as early as thirty minutes after FC, occur in parallel with and as a result of protein synthesis [30]. Therefore, a non-invasive tool that is sensitive to cytoskeletal/cell microstructural changes could potentially identify brain regions most susceptible to fear motivated learning.

*In vivo* magnetic resonance imaging (MRI) has been shown to be a useful probe for cerebral structural alterations that accompany a range of psychiatric disorders [31], [32], [33], [34], [35], including PTSD [36], [37], [38], [39]. We, and others, have shown that parallel MRI differences can be identified in rodent models of psychiatric conditions [40], [41], [42]. Thus MRI may hold promise as a translational tool for investigation of diagnostic and treatment biomarkers in rodent models of PTSD.

Diffusion tensor imaging (DTI) is an MRI technique that can characterize tissue microstructure quantitatively [43], [44], [45], [46]. It has been shown that DTI is sensitive for microstructural alterations in PTSD patients [47], [48], [49], [50], [51]. In addition, recent DTI studies have shown detection of more subtle plasticity changes in human brain using various training paradigms [52], [53], [54]. Animal studies also confirm neuronal plasticity can be probed quantitatively by DTI indices [55],[56],[57]. These studies indicate that DTI can detect long-term neural plasticity weeks to months following relatively extensive periods of training in animals. In a recent DTI study on neuroplasticity in human and rats, learning-induced regional DTI index changes were detected after 2 hours of training [58]. However, a longitudinal study on rapid plasticity within a short period (within 24 hours) after learning has not been carried out. This is important to do because observing the time course of training-evoked changes by neuroimaging methods may help to narrow down candidate mechanisms [59], [60]. Thus we selected FC, which typically occurs over a short timescale (in minutes), as a paradigm for study.

Previous invasive, ex vivo studies have shown that microstructural changes such as dendritic branching, synaptogenesis, and change in dendritic spine density are induced by FC [30], [61], [62]. *In vivo* DTI measurements, and specifically FA, are thought to directly index these tissue microscopic characteristics by describing their directional and voxel-averaged tissue diffusion properties [63], [64], [65]. Therefore in this Proof of Principle study we tested the hypothesis that *in vivo* DTI in combination with voxel-wise analysis would detect changes in brain regions linked to FC as early as one hour following exposure.

## Methods and Materials

### Ethics Statement

All experiments were approved by the Committee on the Use of Live Animals in Teaching and Research (CULATR) at The University of Hong Kong, and were in compliance with the CULATR guidelines for the use and care of laboratory animals (permit number: 2196-10).

### Animals and Behavioral Method

A total of 18 male C57BL/6N mice (90–95 days old) were bred and mated by The University of Hong Kong, Laboratory Animal Unit (LAU). All mice were maintained on a 12 h day/night cycle with access to food and water, and underwent two MRI scans conducted in the light phase. During scanning, mice were anesthetized with a mixture of isoflurane/air (2.5% for induction and 1.5% for maintenance) via a nose cone [66]. Animals were kept warm using a warming pad with circulating water. Respiration rate was consecutively monitored (SA-Instruments, Stony Brook, NY) and kept in normal range throughout the MRI experiments [40].

The FC paradigm involves the association of a neutral environmental cue, the conditioned stimulus (CS), with an inescapable foot-shock, the unconditioned stimulus (US). After a few such pairings, the CS alone elicits physiological and behavioral fear reactions [67], [68]. The experimental setup has been previously described in detail [69]. In brief, on the training day, mice were placed individually into a conditioning chamber (25×25×25 cm^3^) for 6 minutes of habituation where the mice explored the chamber freely. This was followed by 3 paired presentations of a clicker as the CS (30 sec, 4 Hz, 80 dB) and footshock which was applied to the floor grid of the chamber as the US (2 sec, 0.5 mA). The inter-pair interval was 2 minutes with 2 minutes rest after the final clicker/shock pairing in the chamber. The chambers were cleaned with 70% alcohol between each training session.

Standard contextual and cued tests, where mice were re-exposed to the context and explicit cue in the absence of foot-shock, were performed on eight out of eighteen animals one month after FC, following the methods described previously [69]. A video-tracking system EthoVision XT7 (Noldus, Wageningen, The Netherlands) was used for monitoring and recording throughout the training and memory test sessions. The videos were saved for later behavioral analysis [70].

### MRI Protocol

All imaging experiments were conducted using a 7T MRI scanner with a maximum gradient of 360 mT/m (70/16 PharmaScan, Bruker Biospin GmbH, Germany). A quadrature RF coil with 23 mm inner diameter was used. All animals were scanned one day before, one hour after, and one day after FC training. To obtain geometric localization using identical landmark among animals, high resolution anatomical images were acquired in axial, coronal, and sagital views respectively before DTI acquisition. *In vivo* diffusion-weighted (DW) images were then acquired using a SE 8-shot EPI sequence with the following parameters: TR/TE = 3000/28.6 ms, δ/Δ = 5/17 ms, 15 noncollinear gradient directions with a single b-value = 1000 s/mm^2^, and five additional images with b-value = 0 (b_0_ images) [71]. The geometric parameters were: FOV = 2.8×2.8 cm^2^, acquisition matrix = 128×128 (zero-filled to 256×256), 12 slices with 0.48 mm thickness and 0.07 mm inter-slice gap. The diffusion protocol was repeated four times for signal averaging. The DTI data acquisition took 32 minutes, and the entire MRI protocol lasted approximately 50 minutes per animal. This protocol was optimized to minimize geometric distortion, and the fiber directions were confirmed to be unbiased in the color FA maps. In order to ensure schedule of scanning was comparable for every animal, two of the authors worked together to transfer each mouse from FC apparatus to MRI scanner. Transportation between FC test room and the MRI scanner took less than 10 minutes. Following well-practiced routines in our laboratory, induction of anesthesia followed by positioning in the scanner took 10 minutes. Anatomical localization images were acquired over 20 minutes prior to diffusion image acquisition, and all procedures were complete within one hour.

### Data Processing and Analysis

For each animal, diffusion data were first corrected for the eddy current induced displacements using a rigid-body registration to the average of the b_0_ images with A.I.R 5.2.5 [71], [72], [73], [74]. The registered images were screened for motion artifacts using DtiStudio 3.0.2, only images without observable artifacts were used to generate DTI index maps by an in-house written MATLAB program [74]. Fractional anisotropy (FA), mean diffusivity (MD), radial diffusivity (λ_⊥_) and axial diffusivity (λ_//_) maps were calculated by fitting a tensor model to the corrected diffusion data at each voxel using the method described previously [64], [71], [75], [76], [77].

Statistical tests were performed on the repeated measures within the entire group, where each mouse brain volume was normalized to a custom template using a 12-degree-of-freedom affine transformation with 0.1 mm smoothing for transformation parameter estimation. The normalization and statistical procedures were performed using SPM5 (http://www.fil.ion.ucl.ac.uk/~spm/). First, the average pre-FC b0 image from a representative animal was spatially normalized to the corresponding average post-FC b0 image. Then the post-FC b0 image and the normalized pre-FC b0 image were averaged to generate a custom b0 template. The five b0 images from each animal and each time point were averaged and normalized to the custom b0 template. Finally the transformation matrix from each b0 normalization was applied to normalize the corresponding DTI parametric maps (FA, MD, λ_⊥_, λ_//_) respectively. Normalization quality was checked, and fine adjustments were made manually to further improve the normalization accuracy.

Following normalization, voxel-wise t-tests were performed for FA, MD, λ_⊥_ and λ_//_ maps respectively, using a factorial design with two factors: the animal (n = 18) and the time point (with 3 repeated measures) in SPM5. Planned comparisons were designed for hypotheses of a transient or short-term effect. The transient effect shows microstructural changes occurred at 1-hr post-FC but reversed at 1-day post-FC. In this test the contrast weight was (−1 2 −1) or (1 −2 1) on the time point factor. The short-term effect shows increasing or decreasing trend after FC, this was tested using contrast weight (−3 1 2) or (2 1 −3) on the time point factor. For these comparisons, statistical maps with main effects (animal and time point) were obtained with statistical threshold p<0.005 considered to be significant.

The resultant significant clusters from each test were saved as region of interests (ROIs). These ROIs were overlaid to each animal’s DTI index maps at each time point to confirm that the voxels were located on the same region in each brain, and also to further identify their locations with reference to the Paxinos and Watson stereotactic atlas [78].

To examine the FC training effects on DTI quantitation, ROI analysis was performed on the brain structures identified from the statistical tests. For each structure, repeated measures ANOVA was performed with 2 degrees of freedom for time points and 17 (Amg or CG) or 8 (HP) degrees of freedom for mice. Post-hoc tests with Tukey’s multiple comparison test was employed to compare the DTI measurements between different time points using Prism 5.00 (GraphPad Software Inc., California, USA).

For behavioral analysis, percentage of freezing behavior (i.e., absence of movement) throughout the entire FC training, cue test and contextual test sessions were automatically measured using EthoVision XT7 respectively. Data from the first 6 minutes of FC training (pre-shock, free exploring) was compared with those from the later 7.5 minutes of FC training (CS-US pairing period), cue test and contextual test respectively. One-way ANOVA with Dunnett’s multiple comparison test was performed in Prism 5.00 (GraphPad Software Inc., California, USA).

## Results

### Transient Fear Learning Effects (One Hour)

**Figure 1:**
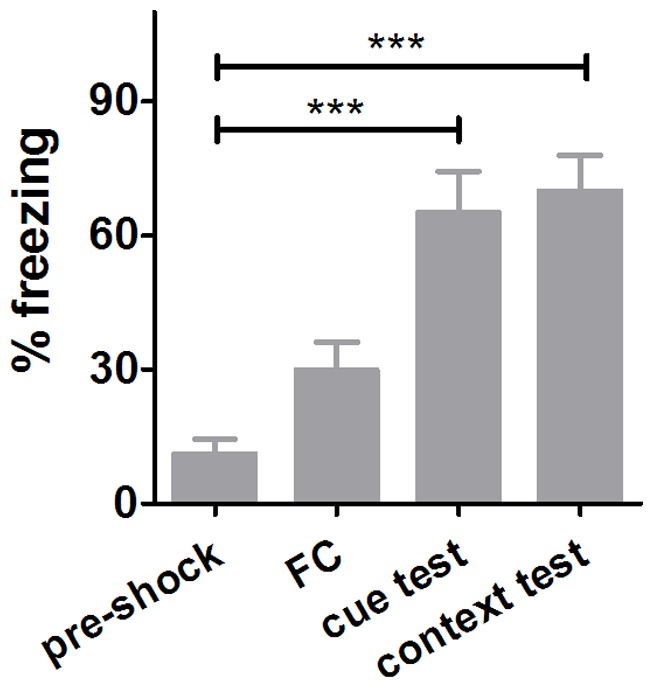
Freezing behavior analysis. Percentage of freezing behavior during pre-shock period (free exploration), FC period (three paired CS with US), and cue/contextual test performed one month post-FC. One-way ANOVA with Dunnett’s multiple comparison test was employed (***p<0.001). Error bars represent the standard error of the mean (n = 8).

Freezing behavior increased during CS-US pairing period compared to pre-shock period in FC training (Fig. 1), confirming that fear-elicited associative learning was quickly acquired. In order to characterize the early effect of fear learning using DTI, planned comparisons were performed. Two slices located posteriorly were excluded in nine out of eighteen animals according to the screening criteria described in methods (due to motion artifacts). Therefore, the sample size for HP analyses (n = 9) was smaller than that for Amg or CG (n = 18).

**Figure 2:**
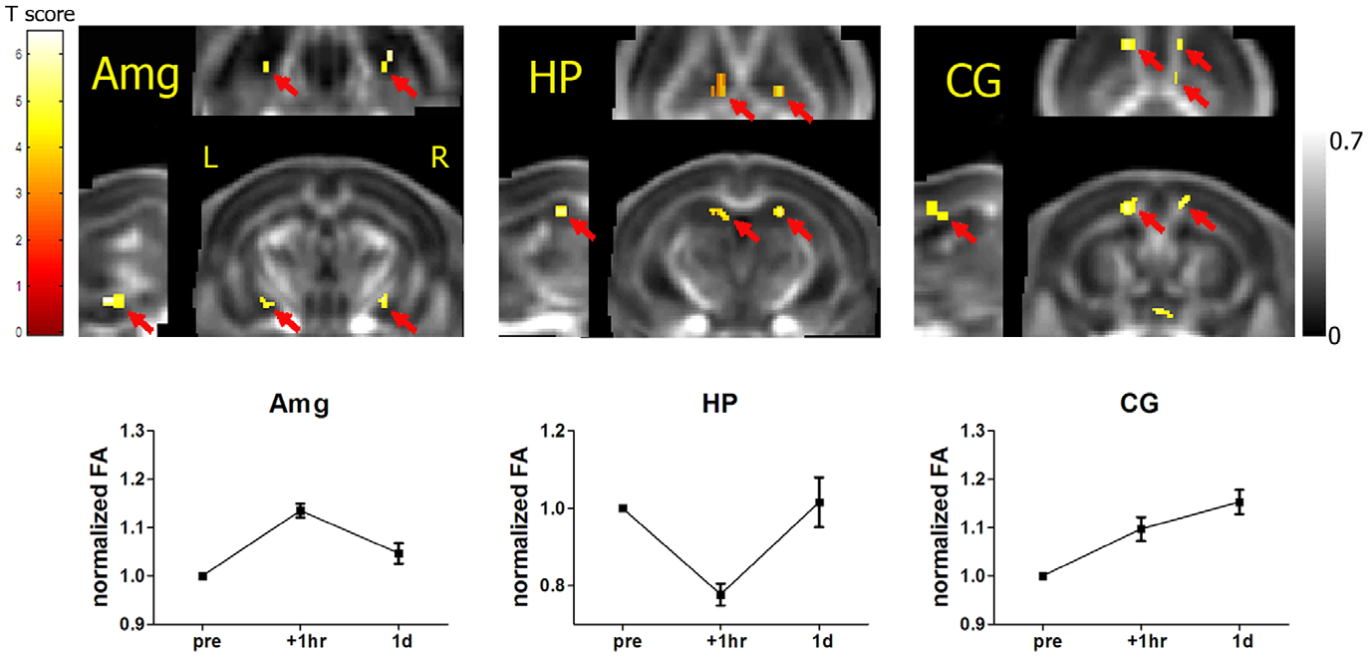
Statistical maps from voxel-wise planned comparisons between time points for FA. Upper row: statistical maps (colored regions) from planned comparisons are overlaid on a mean fractional anisotropy (FA) map (shown in corresponding axial, coronal and sagital views respectively) averaged from all animals. In the clusters indicated by arrows, FA first increased then decreased in amygdala (Amg, p<0.005, 31 voxels, n = 18), first decreased then increased in hippocampus (HP, p<0.005, 33 voxels, n = 9), and kept increasing in cingulum (CG, p<0.005, 49 voxels, n = 18) 1-hr, 1-day post-FC respectively. Colors are coded according to the threshold they exceeded. Lower row: corresponding FA values were presented by normalization of pre-FC at all time points within the significant voxels. Error bars represent mean ± standard error of the mean (n = 18 for Amg and CG, n = 9 for HP).

Significant, but distinct changes in FA were evident in clusters located in amygdala and hippocampi bilaterally (Fig. 2, upper row). In amygdala (Amg, p<0.005, 9 and 22 voxels on the left and right hemisphere respectively), FA first increased at 1-hr post-FC then decreased at 1-day post-FC. Whereas, in hippocampus (HP, p<0.005, 21 and 12 voxels on the left and right hemisphere respectively), FA first decreased then increased. The post-FC FA values were normalized to individual pre-FC FA for each animal in Fig. 2 (lower row), confirming the effect was consistent among animals. There was no significant cluster located in non-CSF brain regions on other DTI indices.

### Short-term Fear Learning Effects (One Day)

In the cue and contextual tests one month post-FC, freezing duration significantly increased compared to that in the pre-shock period (p<0.001, Fig. 1), confirming that fear memory had been established successfully in the trained mice. As shown Fig. 2 (upper row), planned comparison revealed that FA continued increasing among time points in the clusters located in cingulum and adjacent gray matter (CG, p<0.005, 30 voxels on the left anterior hemisphere, 11 voxels on the right anterior hemisphere and 8 voxels on the right posterior hemisphere, n = 18). Post-FC FA values were normalized to individual pre-FC FA for each animal in Fig. 2 (lower row, mean ± standard deviation), confirming this short-term effect was consistent among animals. No significant cluster located in non-CSF brain regions was found in the short-term effect tests on other DTI index comparisons.

### DTI Quantification

**Figure 3:**
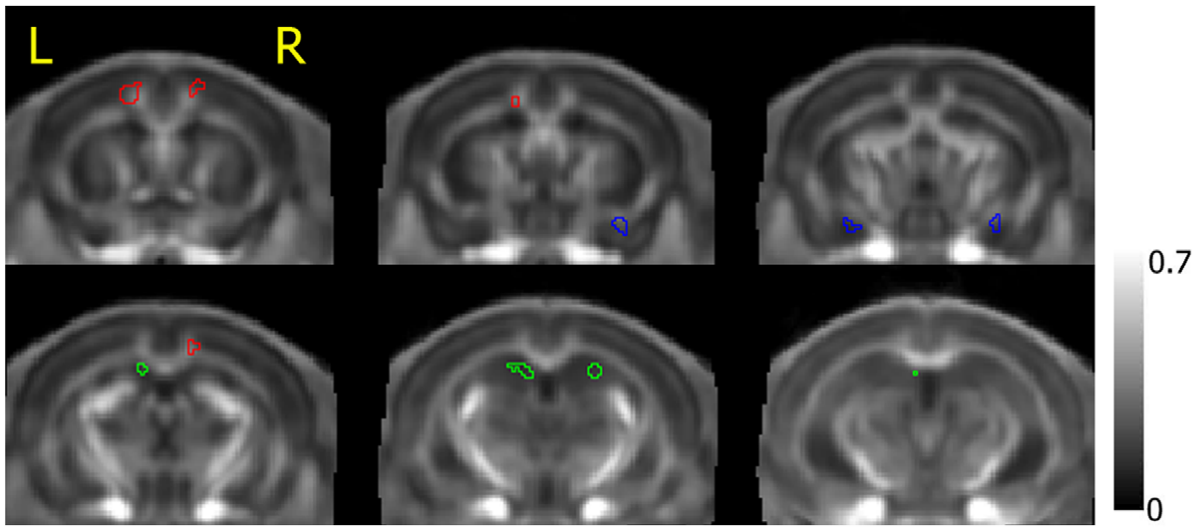
ROI illustration. ROIs obtained from the significant clusters pointed in Figure 2. ROIs were overlaid on an FA map averaged from all animals. Cingulum and adjacent gray matter (red), amygdala (blue) and hippocampus (green) are shown from anterior to posterior (left to right, up to down).

**Figure 4:**
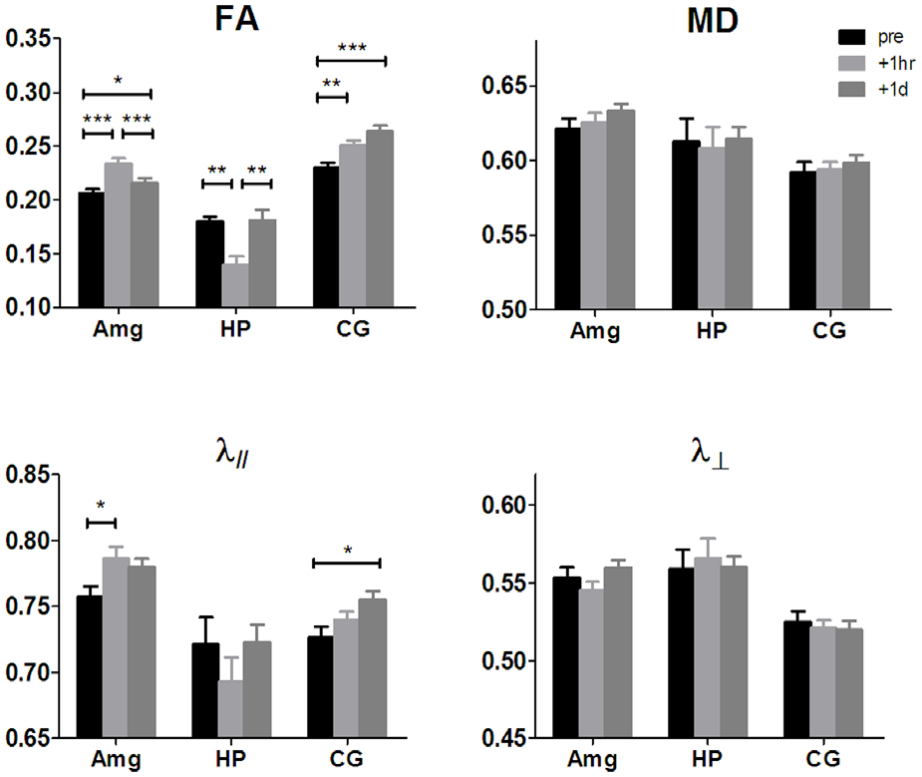
DTI quantification on the ROIs. FA, MD (in µm^2^/ms), radial diffusivity (λ_⊥_ in µm^2^/ms) and axial diffusivity (λ_//_ in µm^2^/ms) between time points were compared using repeated measures ANOVA with Tukey’s multiple comparison test (*p<0.05, **p<0.005, ***p<0.001). Error bars represent standard error of the mean (n = 18 for Amg and CG, n = 9 for HP).

To further examine the effects on each DTI index, the resultant clusters (Amg, HP and CG) from voxel-wise analysis were selected for ROI (shown in Fig. 3) analysis with repeated measures ANOVA. Fig. 4 plotted the DTI index measurements at each time point. There was a significant effect of time on FA measures in Amg (F_2,17_ = 28.54, p<0.0001), HP (F_2,8_ = 12.61, p<0.0005), and CG (F_2,17_ = 19.56, p<0.0001). The effect of time was also significant for λ_//_ in Amg (F_2,17_ = 3.82, p = 0.032) and CG (F_2,17_ = 3.81, p = 0.032). This was explained by an acute increase in FA and λ_//_ in Amg, (13.5±6.1%, p<0.001) and (4.1±7.4%, p<0.05) respectively. In contrast, in HP FA decreased significantly (22.3±8.5%, p<0.01). These changes reversed one day after FC, that is: FA decreased (7.7±6.6%, p<0.001) and λ_//_ decreased (0.7±4.3%, not significant) in Amg; FA increased (31.2±24.7%, p<0.01) in HP. The pattern of FA changes in CG was rather different. FA initially increased by 9.7±10.4% 1-hr post-FC (p<0.01), and continued to increase by a total of 15.3±10.5% (p<0.001) one day post-FC compared to pre-FC. λ_//_ also increased by 4.2±6.9% (p<0.05) one day post-FC compared to pre-FC. There was no significant change in MD or λ_⊥_ across time in any of these three structures. (Percentage change in DTI indices is expressed as mean ± standard deviation).

## Discussion

Combining *in vivo* DTI and voxel-wise analysis, significant FA increase in amygdala and decrease in hippocampus were found bilaterally one hour after FC, and these changes were reversed one day after FC. In addition, increasing FA was observed in cingulum and adjacent gray matter bilaterally one hour and one day after FC. These regions are known to be crucial for FC. Our results therefore provide the first evidence to support the principle that DTI is sensitive to acute and dynamic fear-mediated structural plasticity changes in this rodent model.

### Acute Microstructural Plasticity Following FC

Studies of the time scale of neural plasticity suggest microstructural changes occur rapidly, within hours after training [58], [79], [80]. It has been proposed that synaptic structural modification is mediated by cytoskeletal molecules over hours or days after learning, and these changes contribute to short-term plasticity and memory [28], [81]. Evidence from gene and protein expression studies suggests that neuronal morphogenesis and structural plasticity in amygdala and hippocampus can indeed be observed as quickly as half an hour after FC training [30], [82]. These neuronal alterations have been found to be accompanied by rapid glial-neuron interaction and functional changes [83]. For example, electrophysiological techniques detected an activity peak in the basolateral amygdala 30–50 min after a one-trial fear learning task [84].

Interpretation of DTI findings is intrinsically challenging due to the intricate diffusion properties of complex biological systems and signal averaging in the voxel probed by DTI. Neuronal plasticity processes, such as synaptogenesis and dendritic branching, as well as non-neuronal changes (glial remodeling) such as modification of astrocyte processes are candidate mechanisms underlying learning-induced MRI changes [59]. Although glial remodeling would be expected to reduce the volume fraction of extracellular space in tissue, the microenvironment is also rapidly modulated by active neurotransmission. During astrocytic transformation in particular, astrocytic processes exhibit enhanced motility and directional protrusive activity towards dendrites in a matter of minutes [85]. Synaptic formation can also occur within an hour, and is reversible within hours [85]. Therefore, it is possible that the bulk effect of this early transient plasticity would be to alter the geometry and directional diffusion properties of both intracellular and extracellular spaces.

In addition to FA changes, we observed that axial diffusivity changes were more prominent than MD changes. This disagrees with previous DTI studies of brain plasticity that have reported significant MD changes accompanying FA changes in GM regions [56], [57], [58]. It is possible that the different training protocol timescale (13.5 minutes versus hours to weeks), as well as the cellular responses in the acute and chronic phases post-training, account for this discrepancy [28], [85].

Non-specific vascular changes may also affect acute DTI measurements after learning [86], [87]. However, we reported in our previous study of FA and hypercapnia that, any contribution from vasculature to observed changes in FA distributes globally across both gray and white matter, and induced less than 2% change in FA [88]. In the current study, when voxel-wise planned comparisons were employed, any non-specific/global effect was largely filtered out by using a high statistic threshold.

### Regional Microstructural Plasticity Revealed by DTI

The amygdala has been widely investigated in FC studies [89]. Studies of fear-elicited memory formation have indicated profilin-induced, rapid morphological alterations such as postsynaptic density enlargement and synapse length increase in amygdala [30], [90]. Rapid formation of dendritic spines in motor cortex has also been observed one hour after motor-skill learning revealed by two-photon microscopy imaging [91]. In gray matter, which is mostly constructed of neuronal cell bodies, glial cells, and capillaries, the microscopic environment normally exhibits low anisotropy. It is likely that any enhanced cellular morphological rearrangement that introduces preferential water diffusion direction may contribute to FA and axial diffusivity changes probed by *in vivo* DTI.

For example, during active glial-neuronal interaction, structural modification of astrocyte enhances the motility of astrocytic processes dramatically, and the astrocyte shows directional protrusive activity towards dendrites. Meanwhile, the geometry and diffusivity properties of extracellular space is influenced by such structural modification and become more isotropic. As a result, bulk diffusion anisotropy likely becomes more dominated by the intracellular component with an overall enhanced anisotropy of diffusion environment. In addition, this astrocytic process motility is regulated by diffusible factors from adjacent neuronal elements, and it can be triggered in minutes and is reversible within hours [85]. Extrapolating these findings to our DTI results in amygdala, significant FA changes at 1-hr and 1-day post-FC may reflect these known dynamic interactions of glial cells and neurons, while the enhanced synaptic transmission may play a role in the significant axial diffusivity increase.

The hippocampus holds central importance in PTSD. It is considered to be one of the most “responsive” brain structures due to its rapid plasticity at molecular, cellular, structural, and functional levels. It has also been proposed to be a promising target for PTSD treatment [92]. In this study, the most substantial change observed was FA decrease 1-hr post-FC in hippocampus. This finding was consistent with a recent manganese-enhanced MRI study of a mouse model of PTSD using inescapable footshock, where the authors reported hippocampus volume loss due to a general decrease in axonal structures signaled by a down-regulation of growth-associated protein-43 [93]. Interestingly, our data also showed a clear return to baseline on FA quantitation 24-hr post-FC. In a previous study of axonal reorganization in mice, a net gain of axonal length of 56.5% was observed 5.5 hours after whisker plucking [94]. Supported by this evidence, our data implied that axonal structures may be capable of rapid reorganization after fear learning. In addition, as FC is ‘stressful’, reduced hippocampal neurogenesis and cell survival after acute stress may also affect FA measurements [95], [96]. Therefore, as a result of rapid neuronal alterations, it is possible that local axonal structure remodeling contributed to the significant FA changes detected here.

The cingulum is a collection of white matter fibers allowing communication between hippocampus, amygdala and anterior thalamic nuclei, all of which are involved in emotion formation and processing, learning, and memory. Myelination is known to be modifiable by experience and maturation [83], [97]. An activity-dependent myelination mechanism has been proposed in a recent human study of motor training, where the observed FA change in white matter was accompanied by adjacent gray matter density alterations as well [98]. However, in our short-term dynamic data, the FA increase was attributed to a significant axial diffusivity increase, whereas little radial diffusivity change was detected. Our observations could be partly explained by fiber reorganization [59], such as reduce fiber crossings leading to an ‘enhanced’ connectivity between distributed brain regions triggered by fear learning after FC [99]. Further histological examinations would help to better understand the underlying cellular mechanisms. However, as demonstrated in other and our previous studies [43], [46], [100], diffusion imaging findings reflect complex bulk effects from multiple cellular components (intracellular and extracellular) and their active interactions.

The FA values in hippocampus and caudate putamen observed here were broadly similar to those reported in the literature, with a variation within about 10–20% found in other regions (summarized in Table 1) [101], [102]. Variation across studies could be partly due to differences in ROI definition and the heterogeneous anisotropy within gray matter regions. Differential diffusion parameters used to accomplish different study aims could also play an important role in determining values of DT indices measured. For example, MD measurements reported by Kumar M et al. are higher than those from our study, and this is probably due to the lower b-value = 786.73 s/mm^2^ used by that group. The b-value dependence of diffusivity quantitation has been demonstrated previously [71], therefore taken together, we believe the measurements from the current study are likely to be reliable.

**Table 1:** Comparisons of DTI quantitation measurements between current study and previous studies.

	Hippocampus		Amygdala		Cortex		Caudate putamen		Thalamus	
	Literature [101]	Current study	Literature [101]	Current study	Literature [101]	Current study	Literature [100]	Current study	Literature [101]	Current study
**FA**	0.18±0.03	0.18±0.01	0.25±0.03	0.21±0.02	0.18±0.05	0.16±0.01	0.22±0.01	0.22±0.02	0.23±0.04	0.26±0.04
**MD**	0.75±0.04	0.61±0.05	0.72±0.04	0.62±0.03	0.65±0.12	0.58±0.03	∼0.5	0.58±0.03	0.72±0.03	0.58±0.05

Data for hippocampus and amygdala in current study are the pre-FC measurements shown in Fig. 4, and data for cortex, caudate putamen and thalamus were measured from manually defined ROIs (n = 18).

### Distinct Microstructural Changes in Amygdala and Hippocampus

Evidence for the differential contributions of amygdala and hippocampus to FC was presented by Phillips and LeDoux [103]. Subsequent investigations of the underlying biological process and coordination between component regions of FC neurocircuitry have confirmed distinctly different changes in amygdala and hippocampus. For example, there are reports of gene up-regulation in amygdala and down-regulation in hippocampus after FC [82], and hippocampal neurite loss versus dendritic growth in basolateral amygdala were found in chronic stress models [25], [104], [105]. In parallel, studies of PTSD patients have also found deficient hippocampal function versus exaggerated amygdala responses [10]. In this study, the observation that FA significantly increased in amygdala but decreased in hippocampus, generally agreed with evidence suggesting distinct direction of changes in amygdala and hippocampus following FC.

### Fear Conditioning as a Model of PTSD

Given the diversity of factors influencing the development of PTSD and its range of symptoms, it is unlikely that any single model can fully capture all of its components [13]. With this caveat in mind, FC is still considered a valuable model to study the neurocircuitry and psychobiological mechanisms of PTSD [10], [24]. FC can be translated to PTSD since individuals initially react to a traumatic event (US) with arousal and fear (unconditioned response), and then continue to show arousal (conditioned response) when confronted with trauma-related cues (CS) after trauma [2]. From previous studies using the FC model and PTSD patients, the most consistent finding is the increased activation in amygdala, establishing its pivotal role in fear neurocircuitry [10], [106], [107].

### Conclusions

*In vivo* DTI is sensitive to rapid microstructural changes reflected by FA. We found that FA increased in amygdala and decreased in hippocampus 1-hr post-FC, and it reversed in both regions 1-day post-FC. These imaging findings were consistent with distinct plasticity phenomena in amygdala and hippocampus after FC, and confirmed that measureable dynamic changes occur shortly after FC. In cingulum and adjacent gray matter, FA was increasing in the post-FC time points, suggesting that ‘enhanced’ connectivity in cingulum followed fear learning. As a corollary, we propose that DTI could be explored as a translational tool to capture potential early biological changes in individuals at risk for developing PTSD.
